# Generation and phenotypic characterisation of a cytochrome P450 4x1 knockout mouse

**DOI:** 10.1371/journal.pone.0187959

**Published:** 2017-12-11

**Authors:** Himanshu Kharkwal, Farhat Batool, Frank Koentgen, David R. Bell, David A. Kendall, Francis J. P. Ebling, Ian R. Duce

**Affiliations:** 1 School of Life Sciences, University of Nottingham, Nottingham, United Kingdom; 2 Department of Biochemistry, University of Karachi, Karachi, Pakistan; 3 Ozgene Pty Ltd., Bentley DC, Western Australia, Australia; 4 European Chemicals Agency, Helsinki, Finland; Cornell University, UNITED STATES

## Abstract

Cytochrome P450 4x1 (Cyp4x1) is expressed at very high levels in the brain but the function of this protein is unknown. It has been hypothesised to regulate metabolism of fatty acids and to affect the activity of endocannabinoid signalling systems, which are known to influence appetite and energy metabolism. The objective of the present investigation was to determine the impact of Cyp4x1 on body weight and energy metabolism by developing a line of transgenic Cyp4x1-knock out mice. Mice were developed with a global knock-out of the gene; the full-length RNA was undetectable, and mice were viable and fertile. Both male and female Cyp4x1-knock out mice gained significantly more body weight on normal lab chow diet compared to control flox mice on the same genetic background. At necropsy, Cyp4x1-knock out male mice had significantly greater intra-abdominal fat deposits (P<0.01), and enlarged adipocytes. Metabolic rate and locomotor activity as inferred from VO_2_ measures and crossing of infrared beams in metabolic cages were not significantly affected by the mutation in either gender. The respiratory exchange ratio was significantly decreased in male knock out mice (P<0.05), suggesting a greater degree of fat oxidation, consistent with their higher adiposity. When mice were maintained on a high fat diet, VO_2_ was significantly decreased in both male and female Cyp4x1-knock out mice. We conclude that the Cyp4x1-knock out mouse strain demonstrates a mildly obese phenotype, consistent with the view that cytochrome P450 4x1 plays a role in regulating fat metabolism.

## Introduction

The cytochrome P450 (CYP) 4 family is generally identified with oxygenation reactions involving the ω hydroxylation of saturated, branched chain, unsaturated fatty acids such as prostaglandins (PGs), leukotrienes (LTs), eicosanoids and arachidonic acids (AAs) [1,2]. These reactions have been implicated in the regulation of multiple physiological functions [3,4,5,6]. In mammals, 6 different CYP4 gene sub families are present: CYP4A, CYP4B, CYP4F, CYP4V, CYP4X and CYP4Z [7,8,9,10]. The CYP4X subfamily was identified from EST databases [11], and Cyp4x1 was initially cloned from rat brain [12]. Later, Cyp4x1 was reported in human brain [13] and mouse brain [14]. Cyp4x1 is also highly expressed in the trachea and aorta, which was further confirmed with analysis of EST databases showing significant number of ESTs in brain and aorta [14, 15]. High numbers of Cyp4x1 ESTs were also found in malignant tissues, pointing to a possible role in tumorigenesis [14], and using immunohistochemistry it was shown that Cyp4x1 showed high levels of expression in human breast cancer [16].

The protein coded by the human *CYP4X1* gene is predicted to have 509 amino acids and has 71% nucleotide sequence identity with mouse, 70% with rat, [14], and 75% with dog [13]. The mouse *Cyp4x1* gene is approximately 28 kb and has 12 exons. The role of this orphan cytochrome P450 has been the topic of widespread speculation. The expression patterns in various brain regions and major blood vessels [12–14, 17, 18] suggest a neurovascular function that has been related to the metabolism of arachadonic acid and its derivatives, such as the endocannabinoid anandamide, into hydroxyeicosatrienoic (HETE) and epoxyeicosatrienoic (EET) acids and ethanolamides. These metabolites have been shown to be involved in the regulation of cerebral blood flow [19, 20]. This speculation has been supported by recent evidence of circadian rhythms in expression of Cyp4x1 and production of EETs in the rat brain [21].

We report here the generation of embryonic stem (ES) cell-derived mouse lines lacking a functional Cyp4x1 enzyme as a means to address the functional role of this orphan cytochrome P450. Cyp4x1^Flox/Flox^ and Cyp4x1^Flox/WT^ mouse lines were produced in this study. Cyp4x1^Flox/Flox^ mice were used as a control to examine the Cyp4x1 knockout phenotype, and the Cyp4x1^Flox/WT^ mouse line was used to generate Cyp4x1-knock out mouse lines. In the Cyp4x1 Flox locus, exon 4 was flanked by a PGK-Neo cassette and with *loxP*, leading to global disruption of exon 4 when Cyp4x1^Flox/WT^ mice were mated with UbiCre mice. Both Flox and knock out (KO) mutant Cyp4x1 mice displayed normal viability and fertility, and we provide here an initial characterization of their phenotype. The availability of Cyp4x1 Flox and KO mouse lines should provide a valuable resource to help define the precise physiological functions of Cyp4x1.

## Materials and methods

### Chemicals

Unless otherwise stated all reagents were purchased from Sigma-Aldrich. PCR Master mix (1.1XReady-mix) was from Abgene. Taqman Q-PCR master mix (Gene expression master mix) and cDNA preparation kits (high capacity RNA-to-DNA 4387406) were from Applied Biosystems. Primers and Probes were synthesised by Europhins Genomics.

### Transgenic mice

The strategy employed for generating Cyp4x1-knockout mice was to produce mice in which exon 4 was flanked by *loxP* sequences by homologous recombination using a targeting vector. Following Cre-mediated deletion of exon 4, splicing of exon 3 to exon 5 would cause a frame shift mutation leading to a knock out Cyp4x1 allele. The Cyp4x1^Flox/WT^ mouse line was generated by Ozgene Pty Ltd (Bentley WA, Australia) who also supplied mice with ubiquitous expression of Cre.

#### Cyp4x1 Flox and Knockout mice

Full details of the generation of the targeting vector, breeding and genotyping are presented in Supporting Information (S1 Text and S1, S2, S3, S4 and S5 Figs)

Briefly, the targeting construct (S1 Fig) was electroporated into a C57BL/6 ES cell line, Bruce4 [22]. Homologous recombinant ES cell clones were identified by Southern hybridization (S2 Fig) and injected into albino C57BL/6-Tyr^c-Brd^/NCr (b6-albino) blastocysts. Male chimeric mice were obtained and crossed to C57BL/6J females to establish heterozygous germline offspring on a pure C57BL/6 background. A Cyp4x1^Flox/Flox^ (homozygous floxed) mouse line was produced by cross breeding heterozygous floxed (Cyp4x1^Flox/WT^) mice. The germline mice were crossed to a Cre mouse line to excise Exon 4. Cyp4x1/KO/Cre mice were crossbred to produce homozygous Cyp4x1 null (-/-) or knockout mouse lines. Offspring were genotyped by Q-PCR using sequence specific primers and probes (S3, S4 and S5 Figs). The Cyp4x1 knockout mouse strain has been archived in the European Mouse Mutant Archive EMMA as strain Cyp4x1tmBeld (EM:06896).

### Animal maintenance

Transgenic mice were transferred to Nottingham UK where they were maintained in isolation in the transgenics facility of the BioSupport Unit (BSU). All animal work was approved by the Animal Welfare and Ethical Review Board of the University of Nottingham and authorized under United Kingdom Home Office project license PPL 40/3065. Mice were group housed in single-gender groups after weaning, and maintained at approximately 21°C and 40% humidity. They were allowed *ad libitum* access to water and standard laboratory chow comprising of 18% protein, 44% carbohydrate, 6% fat (Teklad 2018, Harlan, UK). Animals were housed from birth in 12 hours light: 12 hours dark with lights off at 19:00 GMT.

### Cyp4x1 mRNA analysis

Total mRNA was prepared from brain, heart, lungs, aorta, kidneys, liver, spleen, colon and skin of wild type and knockout mice using the Trizol^™^ reagent method. 2μg RNA was reverse transcribed to cDNA and quantified using a Thermo Nanodrop spectrophotometer. Forward (5’ GAAGATATTTCTGAGCAGAA 3’) and reverse PCR primers (5'-TCGATGGTTGTTTCCTG-3') were designed to target the region between exon 3 and 5 in Cyp4x1 mRNA. Primers for the endogenous enzyme GAPDH (forward 5’ AATGGTGAAGGTCGGTGTGAAC 3’ and reverse primers 5’ GAAGATGGTGATGGGCTTCC 3’) were also designed and used as an internal control. A PCR reaction was carried out and amplified products were analysed by agarose gel electrophoresis. The predicted amplicon for WT *Cyp4x1* is 240bps and for the knock out mutant missing exon 4 is 112bps. GAPDH is predicted to produce a 226 bps amplicon [23].

### Body weight and effect of high fat diet

Cyp4x1 knockout mice (12 males and 13 females) and Cyp4x1 Flox controls (8 males and 17 females) were housed in single sex groups of 2–4 littermates and provided with standard laboratory rodent diet and water *ad libitum*. Group food intake in the home cage and individual body weights were monitored weekly from week 4 to week 19. Mean food intake values were calculated after correcting group intake for the number of mice per cage.

To investigate the effect of a high fat diet CYP4x1 knockout mice (8 males and 8 females) and Cyp4x1 Flox controls (7 males and 10 females) were weaned (21–23 days of age) onto standard laboratory rodent diet, (Teklad 2018, Harlan, UK) in single sex groups of 2–4 littermates. At a mean age of 28 days, mice were placed on a high fat diet (45% fat: diet 824018, Special Diet Services, Witham, UK), and body weight and food intake was recorded weekly.

### Comprehensive Lab Animal Monitoring System (CLAMS)

At 14 weeks of age (15 weeks for high fat diet mice) male and female floxed and Cyp4x1 knockout mice (n = 6 per group) were transferred to CLAMS cages (Linton Instrumentation, Linton, UK, and Columbus Instruments, Columbus, OH) for 48 hours. Metabolic parameters (oxygen consumption: VO_2_, carbon dioxide production: VCO_2_, respiratory exchange ratio = VCO_2_/VO_2_ = RER) were calculated using OxyMax v4.2 (Columbus Instruments) and recorded in 9-min bins. Feeding behavior parameters measured included timing and duration of feeding bouts, food intake within a bout (meal size), and total food intake per unit time. Locomotor activity was also measured in 9-min bins, using two sets of infrared beams lining each cage to monitor linear and vertical movement. The system was operated with an air intake of 0.6 L/min per chamber and an extracted outflow of 0.4 L/min. All measurements were taken at an ambient temperature of 21–22°C. Lighting in both the long-term holding room and the room containing the CLAMS was provided by fluorescent strip lighting, which provided about 350–400 lux at the level of the upper row of CLAMS chambers and 250–300 lux for the lower level of chambers.

### Glucose and insulin tolerance tests

After metabolic analysis in the CLAMS mice of both genotypes were fasted overnight and blood samples were collected by cardiac puncture under terminal anaesthesia. Blood glucose was measured using a HemoCue 201 system (Angelholm, Sweden) to establish fasting values in one group of mice. Other groups of mice received an intraperitoneal injection of glucose at 2g/g body weight, and blood samples collected and glucose measured at 15, 30 or 60 minutes post treatment.

In a separate study, fasted mice at 20 weeks of age were either sampled without treatment, or were treated with insulin (1.0U/kg, Actrapid; Novo Nordisk, Denmark) and a blood sample collected under terminal anaesthesia at 30 or 60 min post treatment. Animals were euthanized by injection of pentobarbital sodium (Euthatal: Rhone Merieux, Harlow, UK).

### Epididymal adipose tissue

Cyp4x1-knockout mice and floxed controls (n = 4 per group) were euthanized at 20 weeks of age and epididymal fat pads were removed for further analysis. The wet weight of the epididymal fat pads was measured and tissues were fixed in 2% glutaraldehyde and embedded in epoxy resin. 0.5μm thick sections were cut and stained with toluidine blue. Mean adipocyte diameter was measured in 50 to 100 cells from sections taken from each knockout and control animal using bright-field microscopy and a calibrated eye-piece graticule.

### Statistical analyses

Body weight and home cage food intake data were analysed for effects of genotype and age (2-factor ANOVA with repeated measures) and 24h mean data from metabolic cage studies were analysed for effects of genotype and gender by 2-factor ANOVA (Prism v5.0; GraphPad Software, San Diego, CA). Comparisons of mean epididymal fat pad weights and adipocyte diameters were made using unpaired t-tests (Prism v5.0).

## Results

### Phenotypic characterization of Cyp4x1 Flox and KO mice

Both homozygous and heterozygous floxed and Cyp4x1-knock out mice showed normal viability and fertility. Average litter sizes were not significantly different between strains: Homozygous Flox 5.92 ± 2.8, n = 12 (mean number of pups per litter ± S.D, n = number of litters); Heterozygous Flox 6.33 ± 2.5, n = 9; Homozygous Cyp4x1-knock out 7.35 ± 1.5, n = 14; wild-type 7.33 ± 1.63, n = 6. No embryonic lethality was found either in Flox or in KO mice. The breeding statistics suggest genotypes were distributed according to Mendelian inheritance both in Cyp4x1 Flox and knock out mice (data not shown).

#### Cyp4x1 mRNA analysis

**Fig 1:**
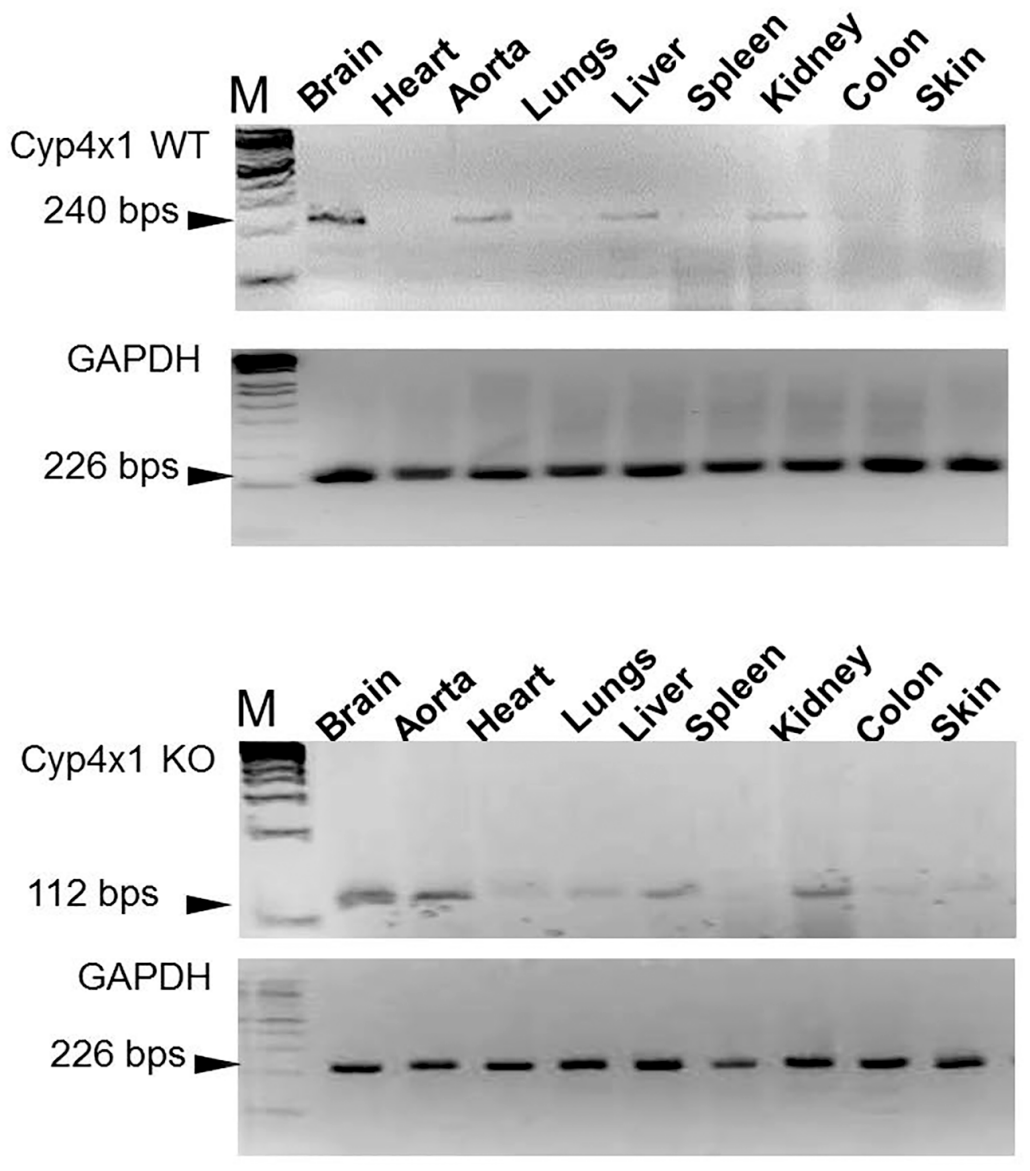
Cyp4x1 mRNA analysis in knock out and wild-type mouse samples. Total RNA was extracted from various tissues and reverse transcribed into cDNA. PCR was carried out using primers targeting the region between exon 3 and 5 of Cyp4x1. The upper panel shows control and knock out Cyp4x1 PCR products from different tissues and the lower panel represents amplification of GAPDH (226bps). The Cyp4x1 control samples produce an amplified product of 240 bps whereas the RNA from knock out mice results in an amplicon of 112bps.

PCR of Cyp4x1 from total RNA resulted in a 240 bps amplicon for the control tissues while in samples from KO mice the same primers produced a 112 bp amplicon (Fig 1) Primers for GAPDH produced the same size (226bp) and intensity bands in samples from both control and KO mice. In addition the different intensity of the Cyp4x1 band in different tissues in control and KO mice (Fig 1) suggests that the level of expression of Cyp4x1 varied between different tissues. Band intensity suggests that Cyp4x1 mRNA was highly expressed in brain, aorta and kidneys in both control and knockout mice, and expressed at lower levels in colon and spleen (Fig 1).

#### Body weight and home cage food intake

**Fig 2:**
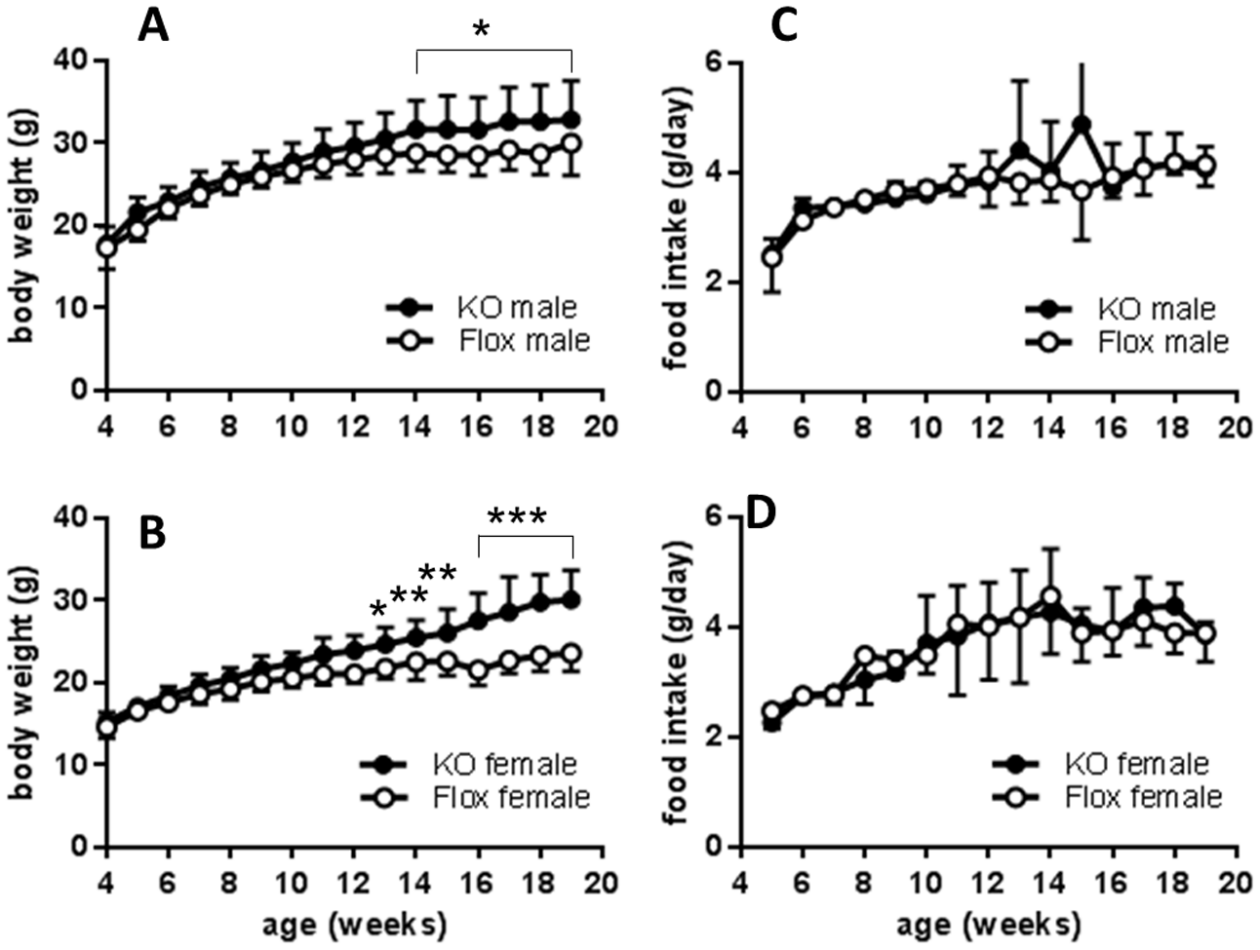
Fig 2 A, B, body weight and Fig 2C, D, food intake in group-housed male and female floxed (○, n = 8 male, n = 17 female) or Cyp4x1 knock out mice (●, n = 12 male, n = 13 female). Values are group mean ± SEM. *p<0.05, **p<0.01 and ***p<0.001 vs floxed controls.

Body weights in KO mice were significantly greater than in control Flox mice after 14 weeks of age in males (genotype x age interaction, P<0.05) and after 11 weeks of age in females (genotype x age interaction, P<0.0001) and continued to diverge until the end of the experiment at 19 weeks of age (Fig 2A and 2B) when male KO mice (32.8±1.3g) and female KO mice (30.1±1.0g) were significantly heavier than their respective Flox controls (males 29.9±1.4g; females 23.6±0.5g). Group mean food intake as measured in the home cage increased with age, but was not significantly different between KO and Flox control mice of either sex (Fig 2D).

**Fig 3:**
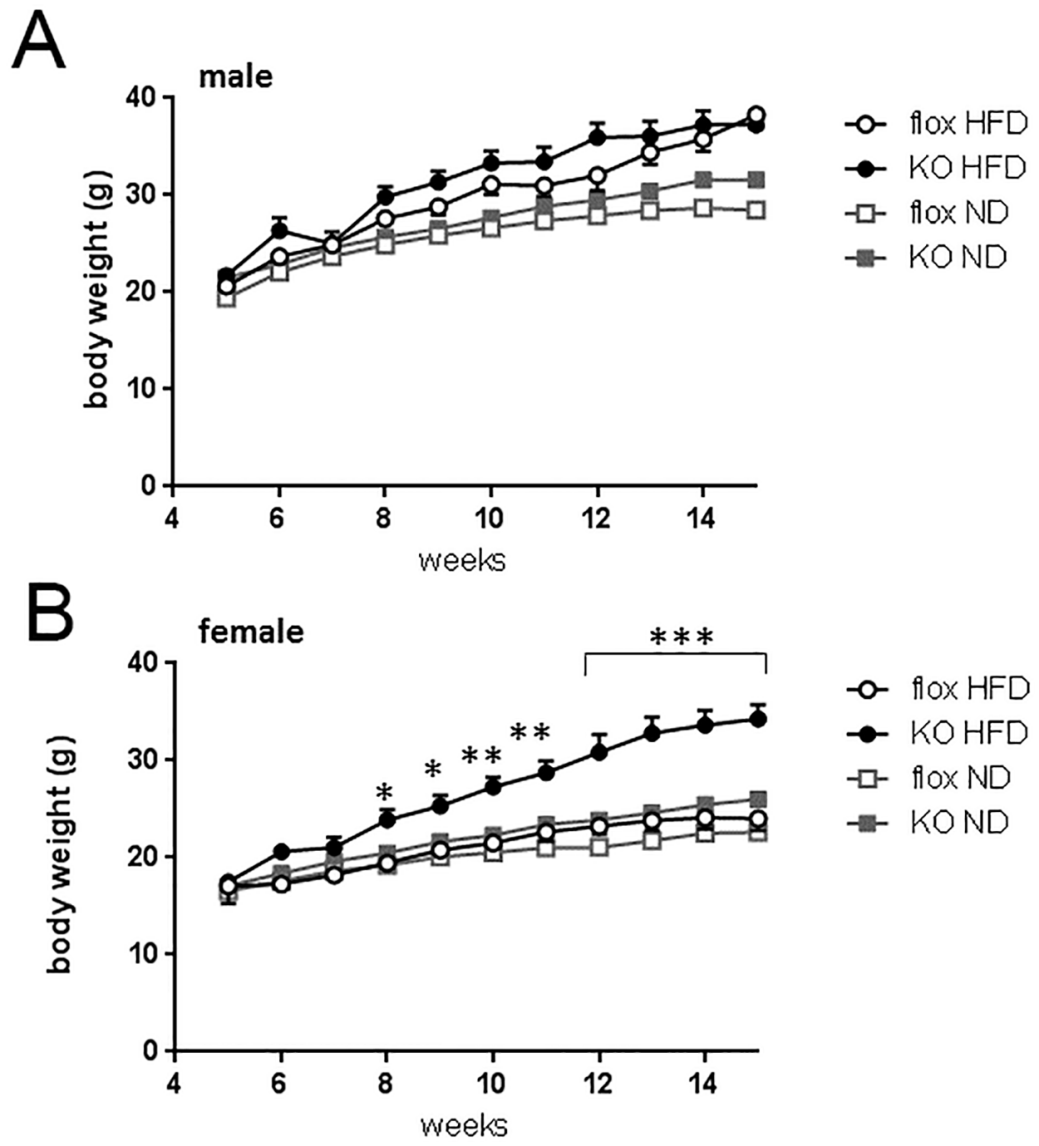
Body weight in group-housed male (Fig3A) and female (Fig3B) floxed (○, n = 7 male, n = 10 female) or Cyp4x1 knock out mice (●, n = 8 male, n = 8 female) kept on high fat diet (45% fat). Values are group mean ± SEM. Body weight for mice kept on normal lab chow (6% fat) are also depicted (flox, knock out data from Fig 2), note the early onset of weight gain in female knock out mice on high fat diet. Body weight *p<0.05, **p<0.01 and ***p<0.001 vs floxed controls.

In male mice, body weight gain on high fat diet was significantly greater than in mice maintained on normal lab chow, but there was no significant effect of genotype on weight gain up to the age of 15 weeks (Fig 3A). In contrast, female Cyp4x1 knock out mice (Fig 3B) gained weight on high fat diet at a significantly greater rate than floxed females (genotype x age interaction, p<0.0001). Post hoc test revealed a significant increase in body weight by 8 weeks of age, that is, 4 weeks after initial exposure to the high fat diet (Fig 3B).

#### Epididymal adipose tissue

**Fig 4:**
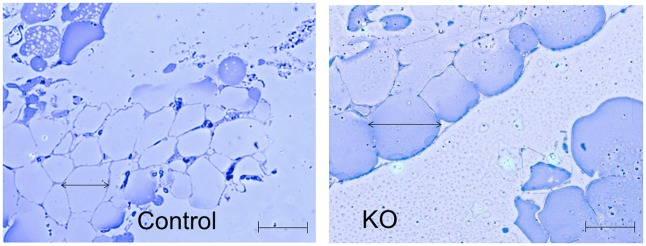
Representative epoxy-embedded sections through epididymal white adipose tissue stained with toluidine blue from a control (left) and a Cyp4x1 knock out mouse (right). Scale bar is 50 μm.

During autopsy it was apparent that KO mice exhibited increased abdominal adipose tissue; the epididymal fat pad weight in KO males (1.67±0.12g) was significantly (P<0.001) greater than in controls (0.51±0.04g). The increase in abdominal adiposity in KO mice appears to be due to hypertrophy of adipocytes (Fig 4), as mean adipocyte diameter within epididymal fat pads significantly increased (P<0.001) from 53.2 ± 2.1 μm to 73.5 ± 2.5 μm.

#### Blood glucose analysis

**Fig 5:**
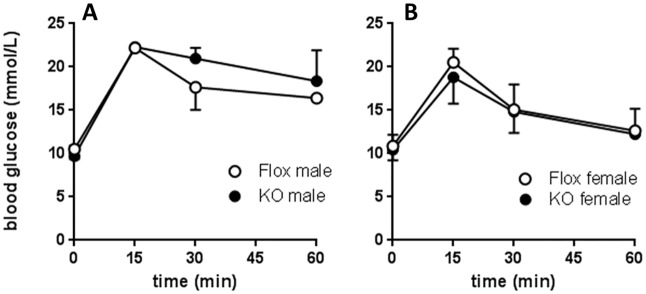
Glucose tolerance test (2g/g BW, ip) in male (A) and female (B) floxed (○) or Cyp4x1 knock out mice (●). Values are group mean ± SEM, group size 3–4 mice per time point.

**Fig 6:**
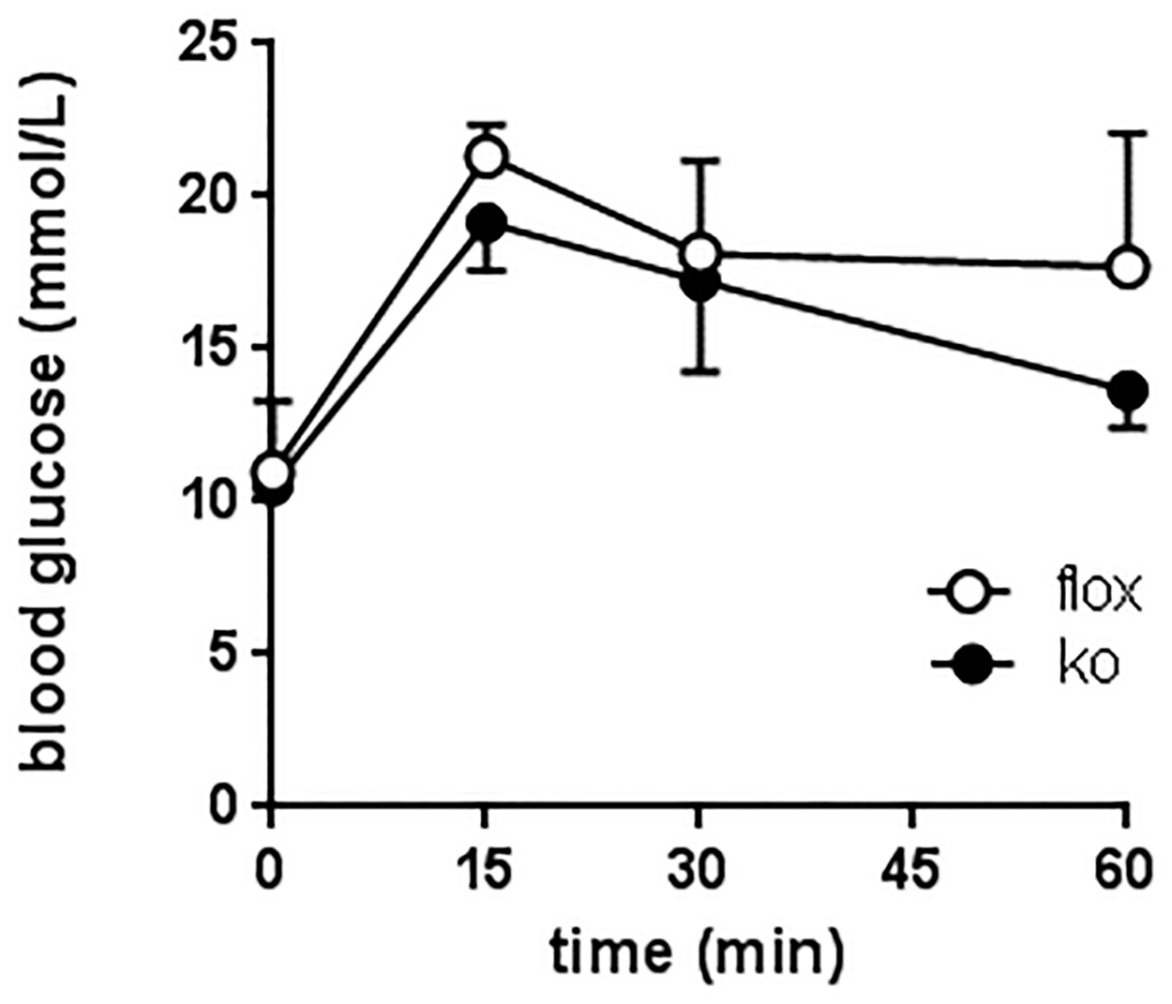
Glucose tolerance test after high fat diet (2g/g BW, ip) in floxed (○) or Cyp4x1 knock out mice (●). Values are group mean ± SEM, group size 2–3 mice per time point.

**Fig 7:**
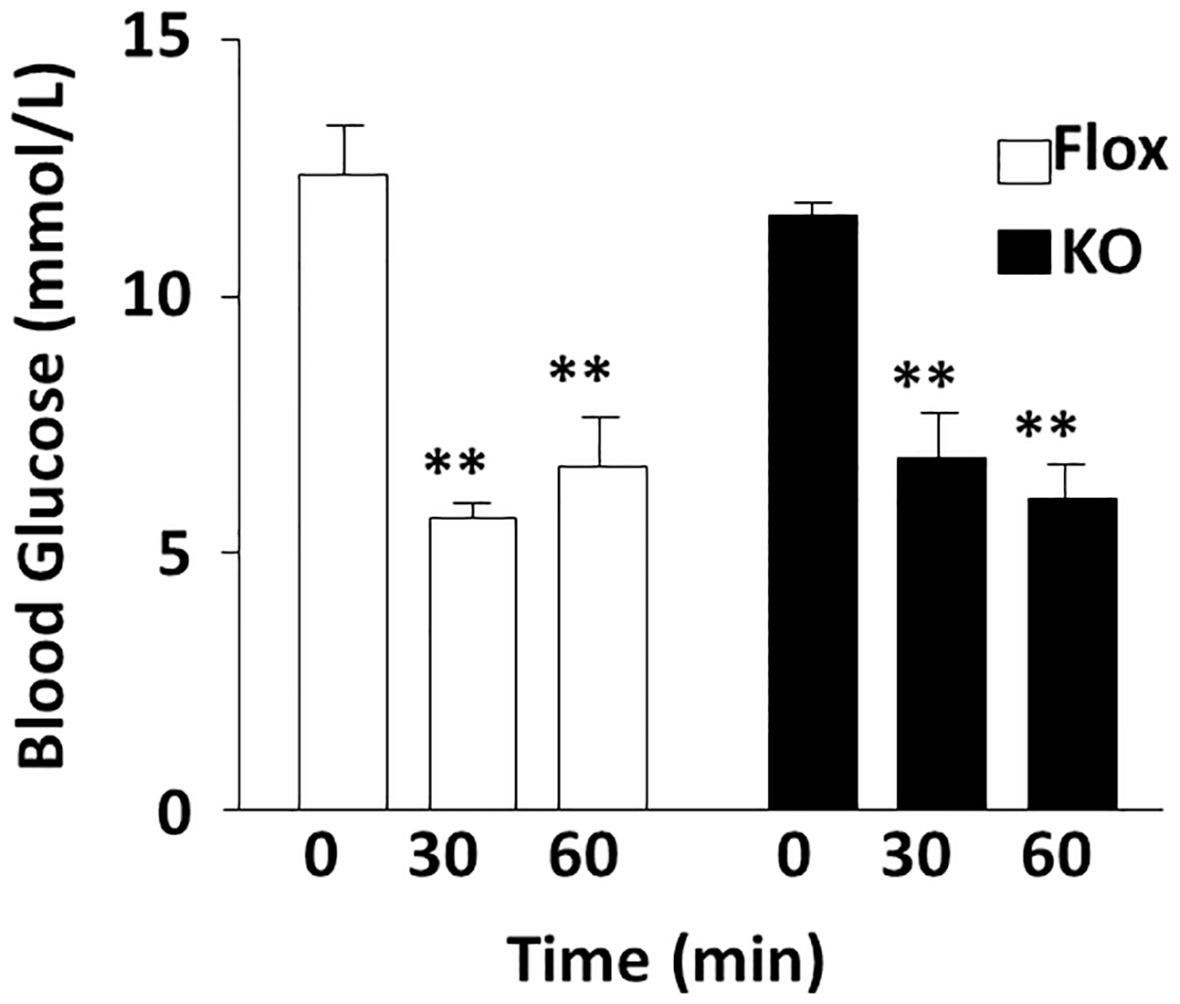
Insulin tolerance test (I IU/kg BW, ip) in floxed (open bars) or Cyp4x1 knock out mice (solid bars). Values are group mean ± SEM, group size 3–4 mice per time point. **p<0.01 vs t = 0.

In order to understand the cause of increased adiposity in Cyp4x1 male KO mice, fasting blood glucose levels and glucose clearance were determined in 20-week-old KO and Floxed control mice. Fasting glucose levels were not significantly different in either male or female KO and Flox control mice, and there was no significant effect of genotype on glucose clearance following (2g/g BW) intraperitoneal glucose loading in mice on a normal diet (Fig 5) or a high fat diet (Fig 6). Insulin tolerance tests were also carried out at 20 weeks of age (Fig 7). In both KO and control mice maintained on a normal diet blood glucose levels were significantly reduced following insulin treatment, but no significant effect of genotype on the response to insulin was apparent (Fig 7).

#### Metabolic parameters

**Fig 8:**
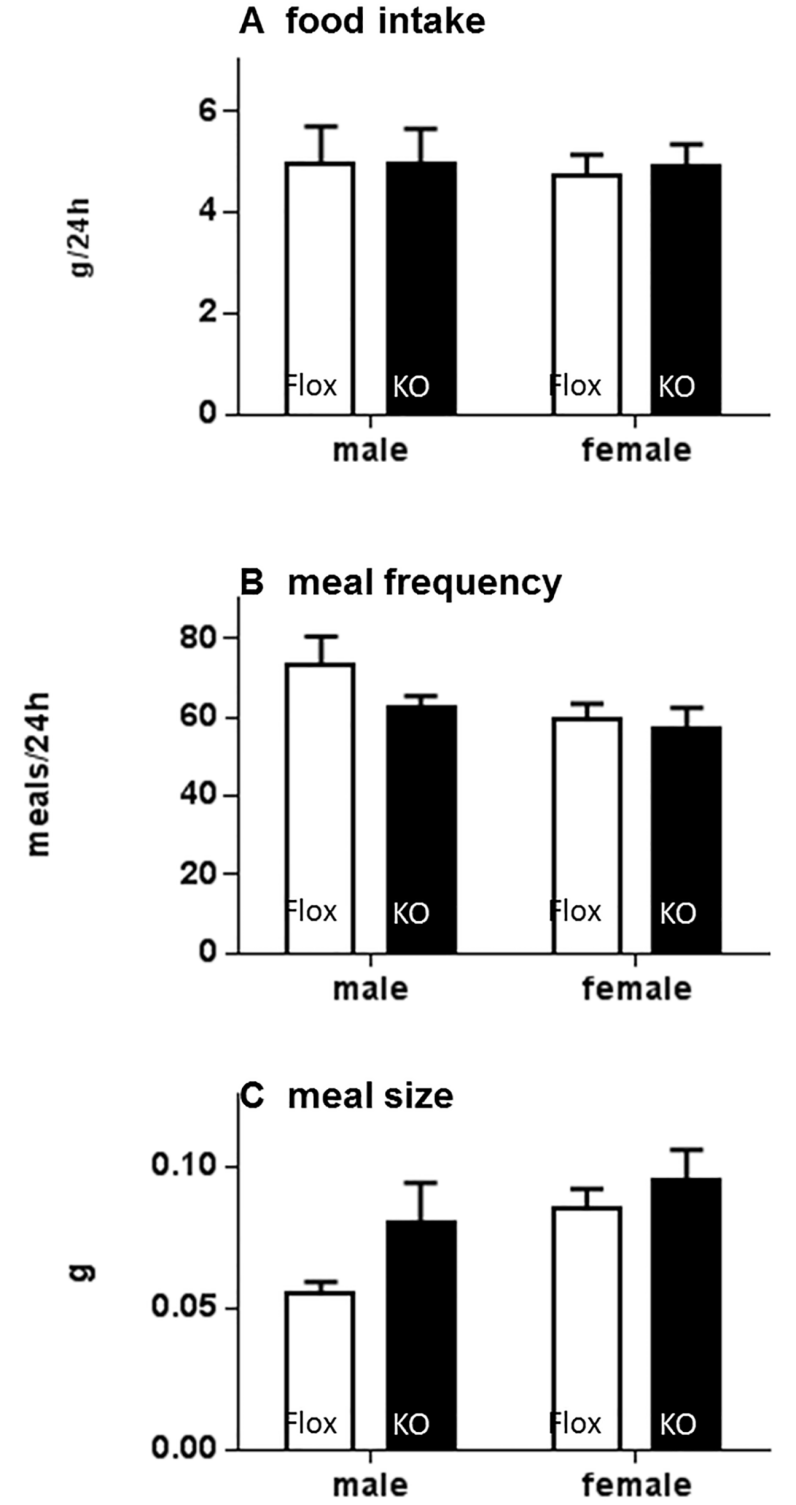
Total food intake (A), meal frequency (B) and meal size (C) in male (left) and female (right) floxed (open bars) or Cyp4x1 knock out mice (solid bars). Values are overall 24h mean ± SEM, n = 6 per group.

**Fig 9:**
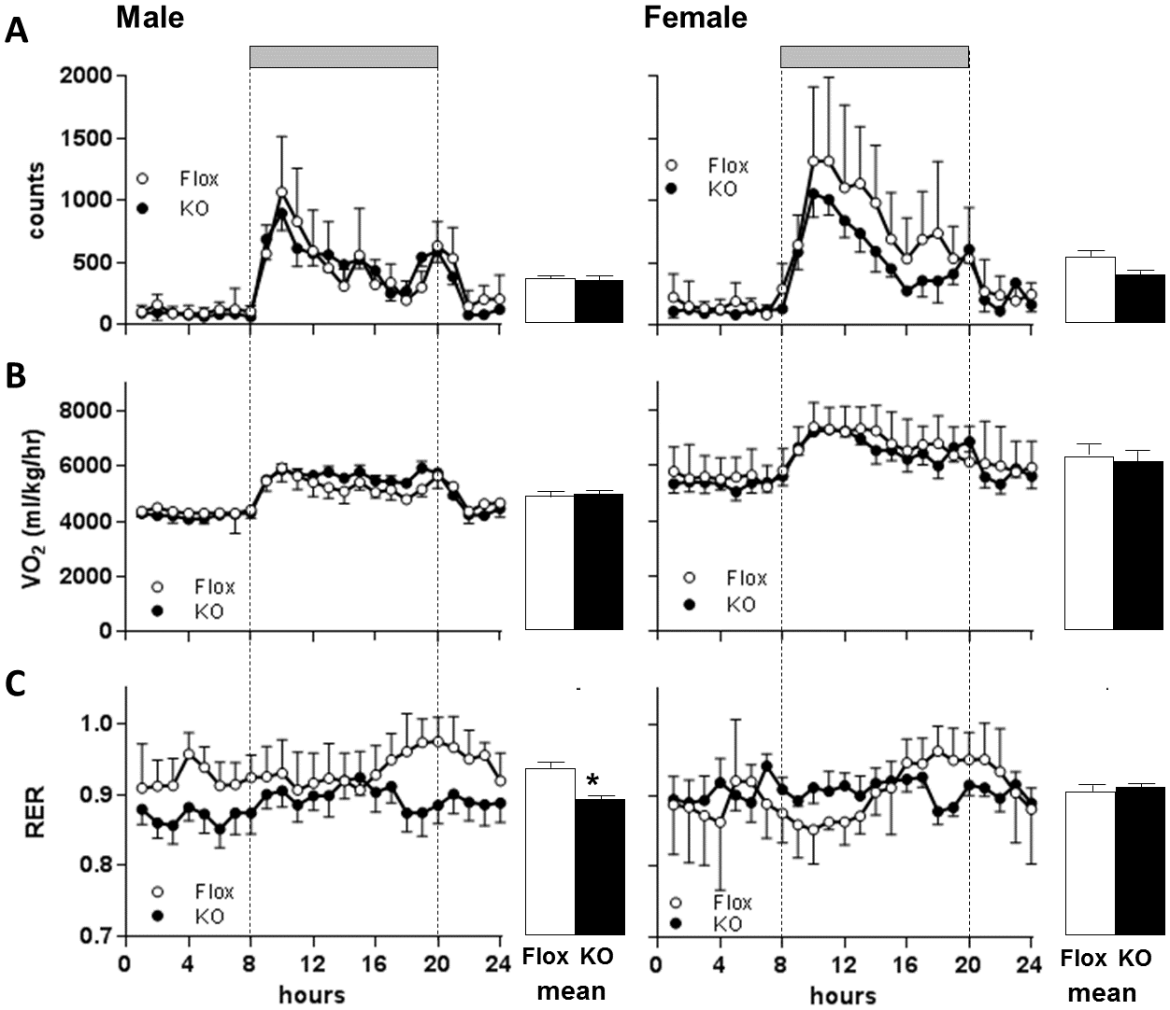
Locomotor activity (A), oxygen consumption (VO_2_ B), and respiratory exchange ratio (RER, C) in male (left) and female (right) floxed (○) or Cyp4x1 knock out mice (●). Grey horizontal bar depicts the dark phase. Values are hourly mean ± SEM, n = 6 per group. Overall 24h mean ± SEM values are also depicted for floxed (open bars) and Cyp4x1 knock out mice (solid bars). *p<0.05 vs floxed controls.

In mice maintained on normal lab chow, no significant effect of genotype on total food intake per 24h or on meal frequency or meal size was observed (Fig 8), consistent with the measures of home cage food intake in group-housed mice (Fig 2). Both Flox and Cyp4x1 KO mice showed clear nocturnal increases in activity (Fig 9A). There was no significant effect of genotype on overall (24h) activity levels in male mice, but there was a trend (P = 0.11) toward decreased activity in KO female mice (Fig 9A). VO_2_ also showed a clear diurnal-nocturnal variation in both genotypes (Fig 9B). Overall VO_2_ was significantly higher in females compared to males (P<0.0001), but there was no main effect of genotype or genotype x gender interaction when mice were maintained on normal lab chow (Fig 9). Analysis of RER values revealed a significant decrease in Cyp4x1 KO males compared to Flox controls (P<0.05, genotype x gender interaction), but no effect of genotype in the females (Fig 9C).

**Fig 10:**
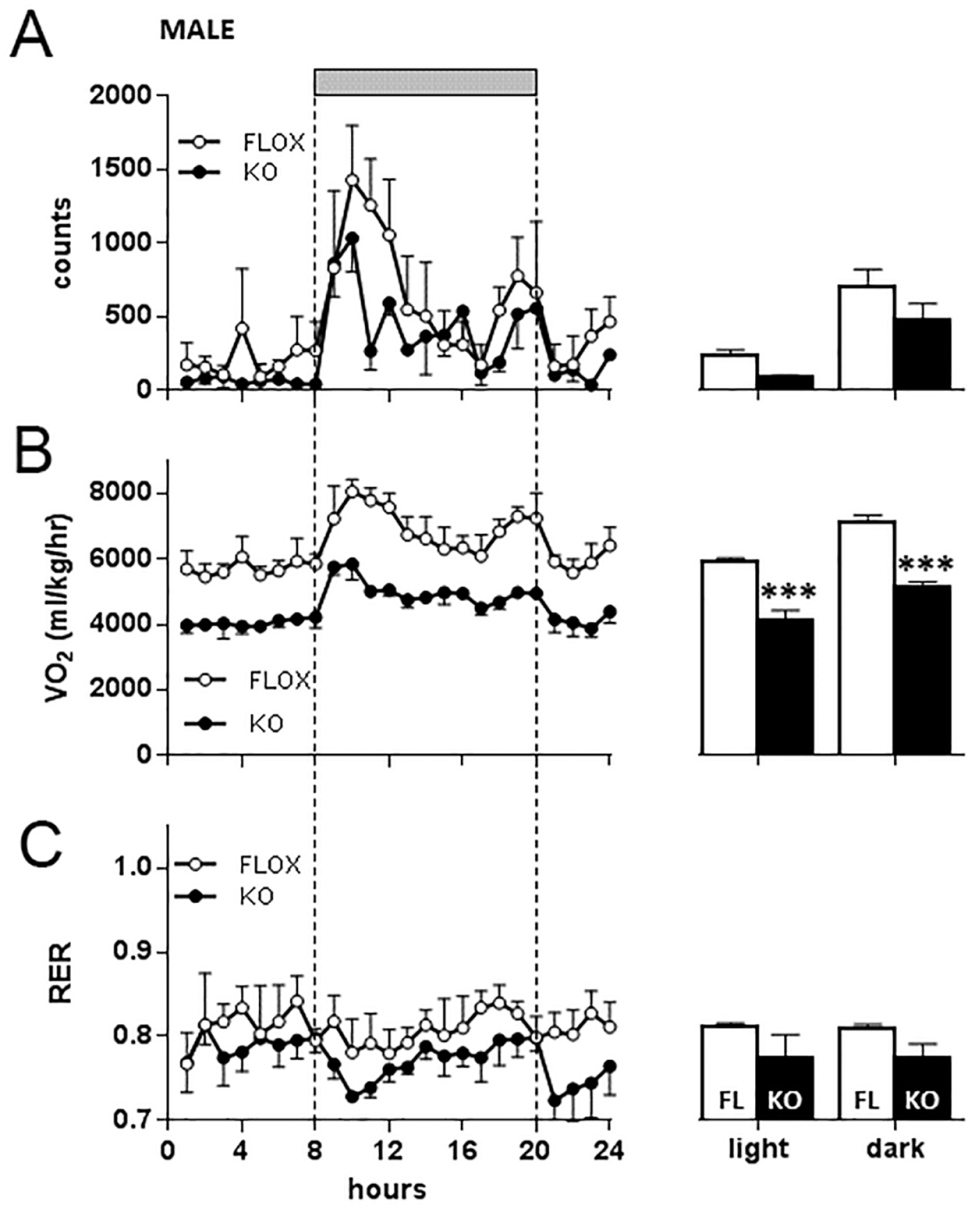
Locomotor activity (Fig 10A), oxygen consumption (VO_2_ Fig 10B), and respiratory exchange ratio (RER, Fig 10C) in male floxed (○, n = 4) or Cyp4x1 knock out mice (●, n = 3). Grey horizontal bar depicts the dark phase. Values are hourly mean ± SEM. Mean (± SEM) values for the light and dark phases are also depicted for floxed (open bars) and Cyp4x1 knock out mice (solid bars). ***p<0.001 vs floxed controls.

**Fig 11:**
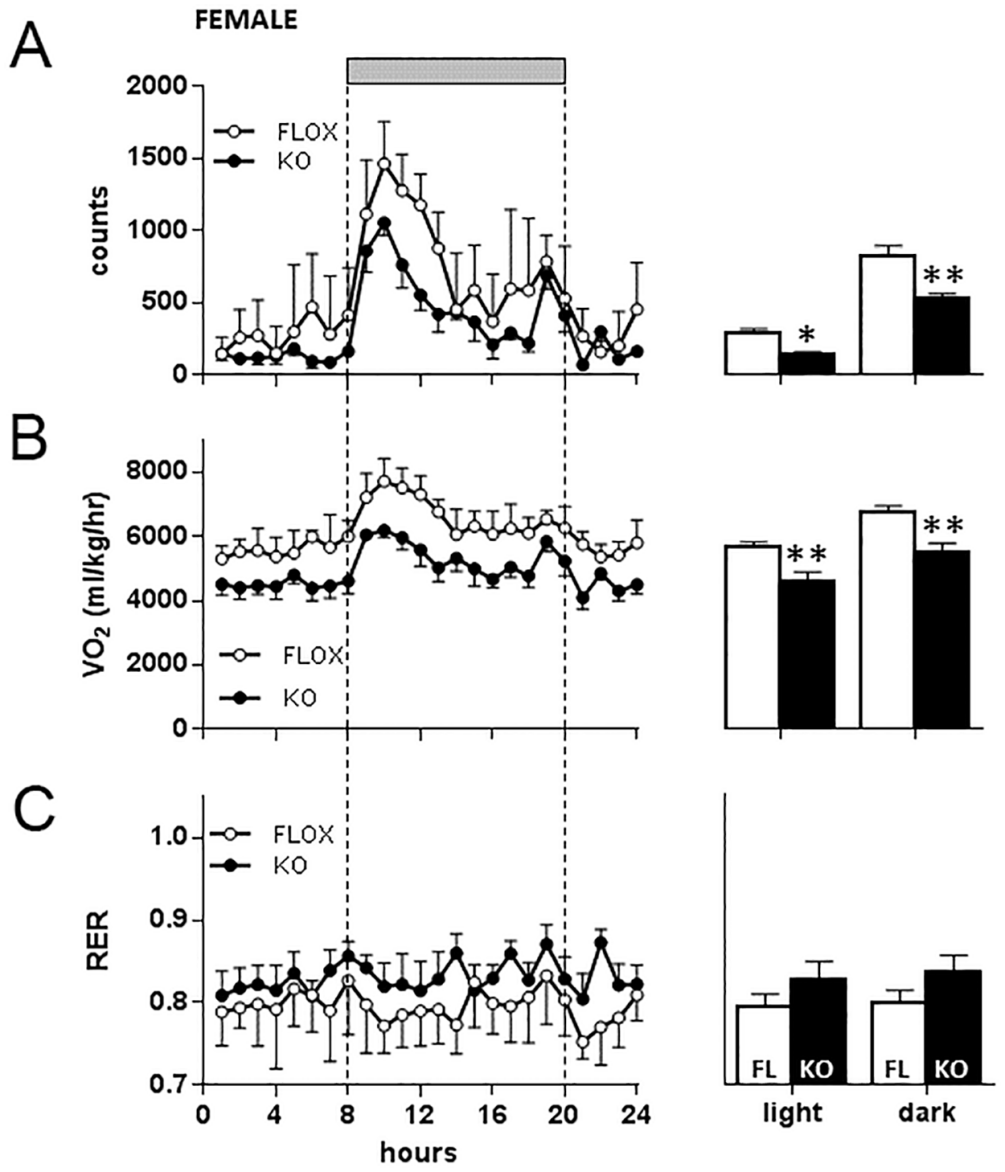
Locomotor activity (Fig 11A), oxygen consumption (VO_2_ Fig 11B), and respiratory exchange ratio (RER, Fig 11C) in female floxed (○, n = 5) or Cyp4x1 knock out mice (●, n = 4). Grey horizontal bar depicts the dark phase. Values are hourly mean ± SEM. Mean (± SEM) values for the light and dark phases are also depicted for floxed (open bars) and Cyp4x1 knock out mice (solid bars). *p<0.05, **P<0.01 vs floxed controls.

In mice maintained on a high fat diet however both floxed and Cyp4x1 knock out male mice showed clear nocturnal increases in activity (Fig 10A), but there was no significant effect of genotype on locomotor activity levels (Fig 10A). VO_2_ also showed a clear diurnal-nocturnal variation in both genotypes (Fig 10B), and there was a highly significant effect of genotype revealed in mice kept on high fat diet, VO_2_ being significantly lower in Cyp4x1 knock out males compared to floxed males during both the light and dark phases (P<0.001, Fig 10). There was no significant effect of genotype or time of day on respiratory exchange ratio in the male mice (Fig 10C). Nocturnal increases in locomotor activity were equally evident in female mice (Fig 11A), but activity levels were significantly decreased in Cyp4x1 knock out females in both the light (P<0.05) and dark periods (P<0.01). There was a clear effect of genotype on VO_2_, being significantly lower in Cyp4x1 knock out females compared to floxed males during both the light and dark phases (P<0.01, Fig 11B). Respiratory exchange ratio in the female mice on high fat diet was not significantly affected by genotype or time of day (Fig 11C).

## Discussion

A Cre/*lox*P system [24] was used successfully to generate a global *Cyp4x1*-null mutation *in vivo*. The *Cyp4x1*-knock out mice were viable and fertile, and no obvious malformations were found in internal organs. Their birth weight did not differ from mice on the same genetic background expressing the floxed Cyp4x1 allele, and growth until weaning was similarly unaffected, but a significant increase in body weight was subsequently observed in both sexes post-weaning. This increased body weight reflected greater abdominal fat deposition, and was more pronounced in females than males. It was late onset, and a relatively modest increase in body weight compared to some other genetic mouse models of obesity, for example the leptin deficient *ob/ob* mouse [25]. When the *Cyp4x1*-knock out mice were tested with glucose or insulin challenges at 20 weeks of age we found no significant impairment of glucose homeostasis, so the phenotype is one of increased adiposity rather than of morbid obesity. This is not a reflection of increased food intake, as measurement of this in both the home cage and in metabolic cages where a detailed assessment of the microarchitecture of ingestion was carried out failed to identify significant effects of the null mutation. When mice were maintained on normal lab chow we found no evidence of increased metabolic rate, as assessed from measures of oxygen consumption, but there was a trend towards decreased locomotor activity in *Cyp4x1*-knock out female mice. Male *Cyp4x1*-knock out mice maintained on lab chow had a significantly lower RER than floxed controls that might reflect an increase in fat oxidation as opposed to carbohydrate oxidation, consistent with their fatter phenotype, but this parameter did not differ significantly in females. However, when mice were raised on a high fat diet a more striking phenotype was observed. Body weight was significantly greater in female mice compared to floxed controls, and energy expenditure (VO_2_) was significantly reduced in both male and female *Cyp4x1*-knock out on a high fat diet compared to floxed controls. To some extent this likely reflects decreased locomotor activity in the *Cyp4x1*-knock out mice, as activity in both the dark and light phases was significantly reduced in female knock out mice on high fat diet. There was also a trend towards decreased diurnal and nocturnal activity in the male knock out mice. There was no effect of genotype on respiratory exchange ratio when mice were housed on high fat diet, but as expected this was substantially lower in all mice on high fat diet as compared to those on standard lab chow, so this would probably mask any additional effect of the *Cyp4x1* mutation. All these parameters were studied across a light-dark cycle as a recent study in Wistar rats [20] found some evidence of diurnal variation in *Cyp4x1* expression in both the brain (hippocampus) and vascular system (inferior vena cava), but we did not detect significant genotype x time interactions that might have indicated phenotypic differences at specific phases of the circadian cycle. The failure to detect clear differences in energy intake or expenditure in *Cyp4x1*- knock out mice when maintained on lab chow raises the possibility that their increased body weight and adiposity reflects increased nutrient absorption from the gut and reduced fecal energy loss, or possibly a reduced efficiency of fat oxidation in the *Cyp4x1*- knock out mice.

This is a somewhat unexpected conclusion because the rationale for investigating the function of the *Cyp4x1* gene was the previous observation by ourselves and others that it is highly expressed within the brain and elements of the vascular system [12,14]. This was confirmed in the current study by tissue-specific PCR analysis of both the wild-type *Cyp4x1* gene and the truncated sequence in the knock out mice. Moreover, we hypothesized that its physiological function would relate to fatty acid signalling in these structures on the basis of its structural similarity to other cytochrome P450 enzymes, which in turn would impact upon appetite control or energy metabolism. This conjecture is reinforced by a recent computational study predicting strong binding of arachidonic acid and anandamide to the Cyp4x1 protein [26]. Our current observations of the phenotype of *Cyp4x1*-knock out mice are more consistent with a peripheral function for this enzyme, so it is notable that we observed strong expression in the liver. Another possibility is that its primary actions are central, but impact upon peripheral function. For example, endocannabinoid signalling in the hypothalamus regulates sympathetic outflow [27], which in turn innervates peripheral fat depots and regulates lipolysis [28]. Thus the loss of central Cyp4x1 activity might impact upon this process, though the observation that respiratory exchange ratio was decreased in male *Cyp4x1*-knock out mice suggests that increased fat oxidation occurs in this genotype, and therefore the increased adiposity is more likely a reflection of increased fat storage than impaired lipolysis.

In summary, we have found that mice lacking a functional Cyp4x1 gene have a mildly obese phenotype that likely reflects impaired fat metabolism or impaired fatty acid/endocannabinoid signalling [13]. As the role of endocannabinoids in control of appetite and energy expenditure remains an area of high interest with regards to the pharmaceutical control of body weight [29], this mouse should provide a valuable model for understanding the specific function of Cyp4x1.

## Supporting information

S1 Text Supporting information generation of a Cyp4x1 knockout mouse. (PDF)

S1 Fig Targeting vector design and disruption of mouse *Cyp4x1* gene. (PDF)

S2 Fig Genotyping of Cyp4x1^Flox/WT^ mice by Southern blotting. (PDF)

S3 Fig Genotyping Cyp4x1^Flox/Flox^ mouse lines. (PDF)

S4 Fig Genotyping of Cyp4x1^WT/KO/Cre^ mice by Southern blotting. (PDF)

S5 Fig Genotyping of Cyp4x1 KO mice. (PDF)
