# Supplementary material for: Generation and phenotypic characterisation of a cytochrome P450 4x1 knockout mouse
Source: PLoS One. 2017 Dec 11;12(12):e0187959. doi: 10.1371/journal.pone.0187959 (PMC5724839; doi:10.1371/journal.pone.0187959)
Supplement: S1 Text — (PDF) [file pone.0187959.s001.pdf]

S1 Text Generation of a Cyp4x1 Knockout Mouse

Generation of the *Cyp4x1* targeting vector

A targeting vector containing a PGK-Neo cassette, which was flanked by a FRT site for subsequent deletion by FLP recombinase, and *loxP* sequences was constructed from three fragments, the 5' homology arm, the *loxP* arm and 3' homology arm, generated by PCR using C57BL/6 mouse genomic DNA and the primers shown in Table 1. The 6.7 Kbps 5' homology arm contains *NheI* and *NotI* restriction sites; the 761 bps *loxP* flanking arm contains *PacI* and *NheI* sites and

Target	Sequence (5'➔3')
LoxP-flanked arm Forward	CAGCTAGCAGATATCAGGTACCAGACAAAAGTCAT AACTTCGTATAGCATACATTATACGAAGTTATTCAG GCCTCAAAGCCTTCACTTTC
LoxP-flanked arm Reverse	CAGCTAGCAGGATCCAGATATCTTAATTAAGCCTCA AACCTTTTCTCACATCACTC
5' homology arm (5H) Forward	CAGCGGCCGCACCACAAATTGCAGCCTGCCATTG
5' homology arm (5H) Reverse	CAGCGGCCGCTAGCTAAGCCTTAGCTTCACAGTGAC GGAG
3' homology arm (H3) Forward	CATACGTAGCGGCCGCGAAATGGACTTGGCTGATGG GTATG
3' homology arm (H3) Reverse	CATACGTAGGAGGACATTATGCAAAGCAACTCG

Table 1. PCR primers used to generate the targeting vector

finally the 3' homology arm of 6.4 Kbps contains a *NotI* site for cloning into the pBAD vector (Invitrogen) to generate the complete targeting vector. The targeting vector was analysed by restriction analysis and by direct sequencing and the correct

sequence confirmed. The targeting vector was linearised with *Pvu I* and a schematic of the complete targeting vector design and knockout *Cyp4x1* genomic locus is shown in S1 fig.

Generation of *Cyp4x1* Flox and Knockout mice

The *Pvu I* linearized targeting vector was electroporated into Bruce 4 ES cells (Millipore).

Recombinant clones were selected on G418 antibiotic medium and correctly targeted clones were identified by Southern blotting following digestion with *EcoRV* and the 3' probe which produces a band of 32.6Kb for the wild-type and 12.7Kb for the floxed locus.

ES cell clones, positive for (Cyp4x1 $^{Flox/WT}$) were selected for expansion. ES cells were microinjected into blastocysts derived from albino C57BL/6-Tyr^{c-Brd}/NCr (b6-albino) female mice and then transferred into recipient CBB6F1 mice (generated by crossing C57Bl/6 male mice with CBA females) for production of chimera offspring. Chimeric mice were backcrossed with C57BL/6 mice to generate F1 mice that were heterozygous for the Cyp4x1-Flox allele (Cyp4x1$^{Flox/WT}$). Genomic DNA from the tail was digested with *KpnI* and probed with the *enP* probe. The genotype of the F1 heterozygous mice was confirmed by Southern blotting where the wild-type allele produces a 10kb band, the Cyp4x1^{Flox} allele produces a 4.3kb band and heterozygote Cyp4x1$^{Flox/WT}$ digests produced both bands (S2 fig.).

A Cyp4x1$^{Flox/Flox}$ (Homozygous floxed) mouse line was produced by cross breeding heterozygous floxed (Cyp4x1$^{Flox/WT}$) mice. Offspring were screened by qPCR using Cre, WT and KO sequence specific Taqman probes (Table 2). For genotyping, 25 µl

of Gene PCR master mix™ (Applied Biosystem) and 10 ng of template DNA were used per reaction. A standard 2 hour reaction was set up each time and used the standard comparative C_T ($\Delta\Delta C_T$) method for quantification. The following method was used for the qPCR reaction cycle; a holding stage at 95°C for 10 minutes and cycling stage of 40 cycles at 95°C for 20 sec, 56°C for 1 minute 20 sec, and 72°C for 30 sec. All genotyping results were analysed by Applied Biosystem 7500 v2.0.4 software and the results were confirmed by gel electrophoresis (S3 fig.) providing a reliable and accurate method of genotyping the mouse colony.

Table 2: List of PCR primers and probes used for genotyping of Cyp4x1 Flox and Knockout mice.

Target	Sequences
Cre (F)	5' ATATCTTCAGGCGCGCG 3'
Cre (R)	5' TTCCATGAGTGAACGAACCTG 3'
Cre (Probe)	5' FAM-AGCTAAACATGCTTCATCGTCGGTCCG-BHQ1 3'
Cyp4x1 WT (F)	5'TTGTATGTGTGCCTTCATGGA 3'
Cyp4x1 WT (R)	5'CAGCTCTGATGCTCACACCA3'
Cyp4x1 WT (Probe)	5'HEX-AGCTAAGGCTTATCAGGCCTCAAAGCC-BHQ1 3'
Cyp4x1 Flox (F)	5' TTGTATGTGTGCCTTCATGGA 3'
Cyp4x1 Flox (R)	5' CAGCTCTGATGCTCACACCA 3'
Cyp4x1 Flox (probe)	5'CY3-ACATTATACGAAGTTATTCAGGCCTCAAAGCC-BHQ2 3'
Cyp4x1 KO (F)	5'TTGTATGTGTGCCTTCATGGA 3'
Cyp4x1 KO (R)	5'TACCCATCAGCCAAGTCCAT 3'
Cyp4x1 KO (Probe)	5'CY5-GTTATGGATCAGGCGCGCCTTT-BHQ2 3'

Heterozygous Cyp4x1$^{Flox/WT}$ mice were mated to a mouse with ubiquitous Cre expression (CreΔneo/CreΔneo). The aim of this mating was to produce double heterozygotes (Cyp4x$^{Flox/WT,Cre/WT}$). Crossing of the double heterozygotes should result in some animals where Cre recombinase acts on the two *loxP* sites flanking the Cyp4x1 locus to excise the floxed DNA fragment including exon 4 to produce a Cyp4x1 null allele.

Pups produced from this mating were genotyped by Southern blotting using the *enP* probe (S4 fig.). Genomic DNA from the tail was digested with *KpnI* and probed with *enP*. An extra band at 1.8Kbs (S4 fig. (4A, lane 1)) represents the Cyp4x1 knockout locus ((S4 fig. (4B)) identifying this mouse as Cyp4x1/KO/Cre. The membrane was re-probed with Cre and neo1 probes in order to confirm the genotype (data not shown).

A colony was established by cross breeding Cyp4x1/KO/Cre mice to produce a homozygous Cyp4x1 null (-/-) or knockout mouse line. *Cyp4x1* and *Cre* genes were not linked hence breeding produced various genotypes including a *Cyp4x1* knockout. A similar q-PCR assay to that described above for genotyping of the crossbred mice produced. Genotypes were confirmed by gel electrophoresis (S5 fig.), where sample 5 represents the Cyp4x1 Knockout.

The Cyp4x1 knockout mouse strain has been archived in the European Mouse Mutant Archive EMMA as strain Cyp4x1tmBeld (EM:06896).

S1 Fig: Targeting vector design and disruption of mouse *Cyp4x1* gene. S1_fig.pdf

S2 Fig: Genotyping of Cyp4x1$^{Flox/WT}$ mice by Southern blotting. S2_fig.pdf

S3 Fig: Genotyping Cyp4x1$^{Flox/Flox}$ mouse lines. S3_fig.pdf

S4 Fig: Genotyping of of Cyp4x1$^{WT/KO/Cre}$ mice by Southern blotting. S4_fig.pdf

S5 Fig: Genotyping of Cyp4x1 KO mice. S5_fig.pdf
