# Supplementary material for: Generation and phenotypic characterisation of a cytochrome P450 4x1 knockout mouse
Source: PLoS One. 2017 Dec 11;12(12):e0187959. doi: 10.1371/journal.pone.0187959 (PMC5724839; doi:10.1371/journal.pone.0187959)
Supplement: S1 Fig — (PDF) [file pone.0187959.s002.pdf]

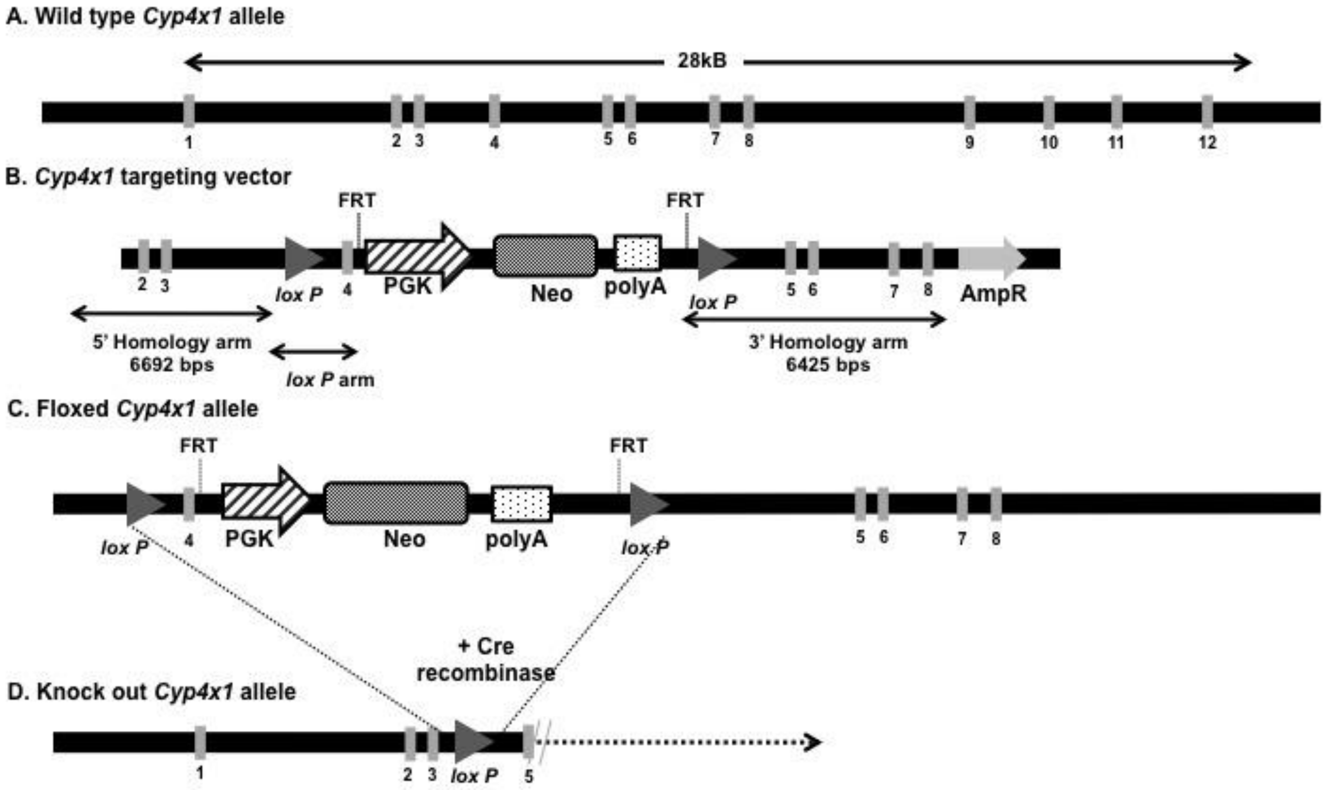

S1 Fig: Targeting vector design and disruption of mouse *Cyp4x1* gene. Figure A-D represents the map of a wild-type *Cyp4x1*allele (A) , a targeting vector (B), Floxed allele (C) and a Knockout *Cyp4x1* allele (D). Approximately 6.5KB fragments both from 5' and 3' mouse *Cyp4x1* gene homology were cloned along with *loxP* into a replacement type targeting vector (B). One *loxP* site was cloned upstream of exon 4 and second into intron 4 along with PGK-Neo selection cassette. *LoxP* sites are indicated by *triangles*, and the selectable cassette (PGK-*neo*) is indicated by the hatched area. This replacement type vector was then used for homologous recombination in Bruce 4 ES cells and as a result a Floxed allele was produced (C). When Cre recombinase acts on *LoxP* sites, it removes the sequence in between two lox P and hence produce a disrupted or KO *Cyp4x1* as a result of frame-shift mutation (D).
