# Supplementary material for: Generation and phenotypic characterisation of a cytochrome P450 4x1 knockout mouse
Source: PLoS One. 2017 Dec 11;12(12):e0187959. doi: 10.1371/journal.pone.0187959 (PMC5724839; doi:10.1371/journal.pone.0187959)
Supplement: S2 Fig — (PDF) [file pone.0187959.s003.pdf]

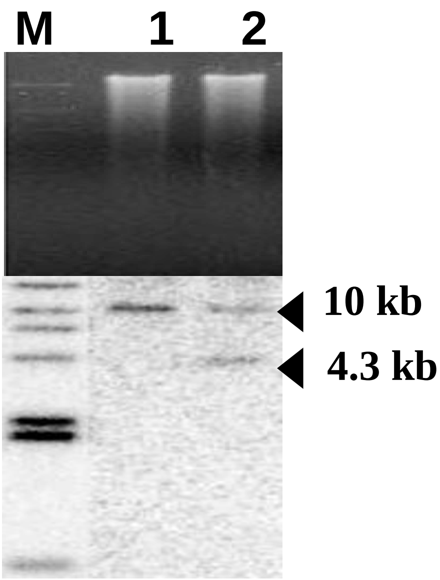

S2 Fig: Genotyping of Cyp4x1$^{Flox/WT}$ mice by Southern blotting.
Litters produced from chimera and female C57BL/6 albino matings were genotyped by Southern blot analysis. Genomic DNA was digested with *KpnI* and probed with the *enP* probe. The wild type allele produces a 10.0 kbp band (lane 1) and the Cyp4x1^{Flox} allele produces an additional 4.3 kbp band (lane 2).
