# Supplementary material for: Generation and phenotypic characterisation of a cytochrome P450 4x1 knockout mouse
Source: PLoS One. 2017 Dec 11;12(12):e0187959. doi: 10.1371/journal.pone.0187959 (PMC5724839; doi:10.1371/journal.pone.0187959)
Supplement: S3 Fig — (PDF) [file pone.0187959.s004.pdf]

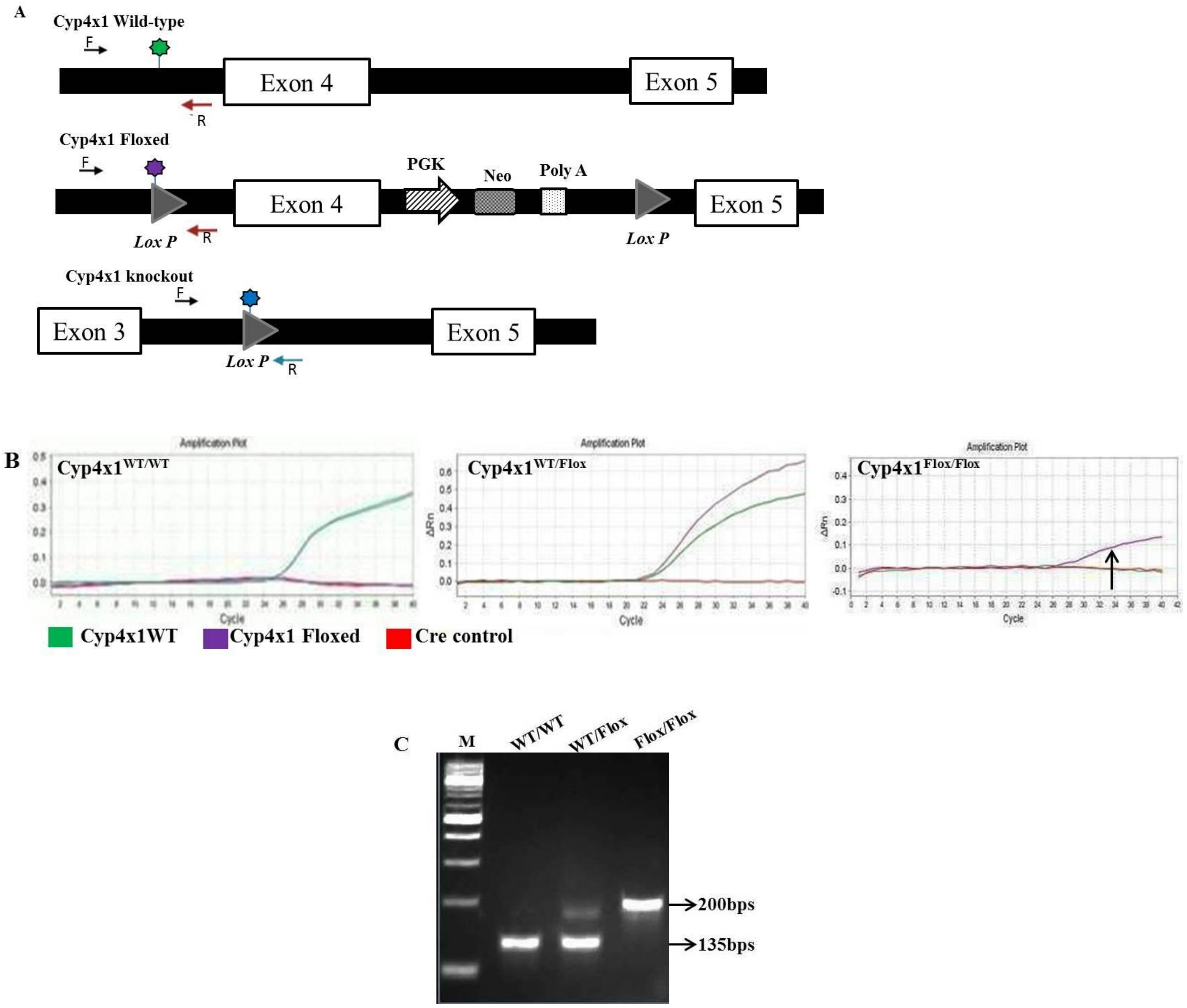

S3 Fig: Genotyping Cyp4x1$^{Flox/Flox}$ mouse lines.

Forward and reverse primers target the intron between exon 3 and 4. The same sets of primers were used for both the Cyp4x1^{WT} and Cyp4x1^{flox} alleles with 2 different taqman probes for wildtype (green) and flox (purple) targets (figure 3A). A mouse with Cyp4x1$^{WT/WT}$ alleles produces a 135 bp amplicon and a signal with the green wild-type probe (Figure 3B left panel) and the homozygous Cyp4$^{Flox/Flox}$ allele will produce a 200bps amplicon with the same primers and only give a signal with the floxed (purple) probe (Figure 3B right panel). If the mouse is heterozygous Cyp4x1$^{Flox/Wt}$ it produce 2 bands with the same primers, 1 at 135bps and 1 at 200bps, and will give signals with both the wildtype and the floxed probe (figure 3B middle panel). Genotypes were re-confirmed by agarose gel electrophoresis of Q-PCRed samples(figure 3C). Lane 1 contains the Cyp4x1$^{WT/WT}$ (a band of 135 bps), lane 2 contains the heterozygous Cyp4x1$^{Flox/WT}$ genotype, where 2 bands were produced; Lane 3 represents the homozygous Cyp4x1$^{Flox/Flox}$ genotype with a single band at 200bp.
