# Supplementary material for: Generation and phenotypic characterisation of a cytochrome P450 4x1 knockout mouse
Source: PLoS One. 2017 Dec 11;12(12):e0187959. doi: 10.1371/journal.pone.0187959 (PMC5724839; doi:10.1371/journal.pone.0187959)
Supplement: S4 Fig — (PDF) [file pone.0187959.s005.pdf]

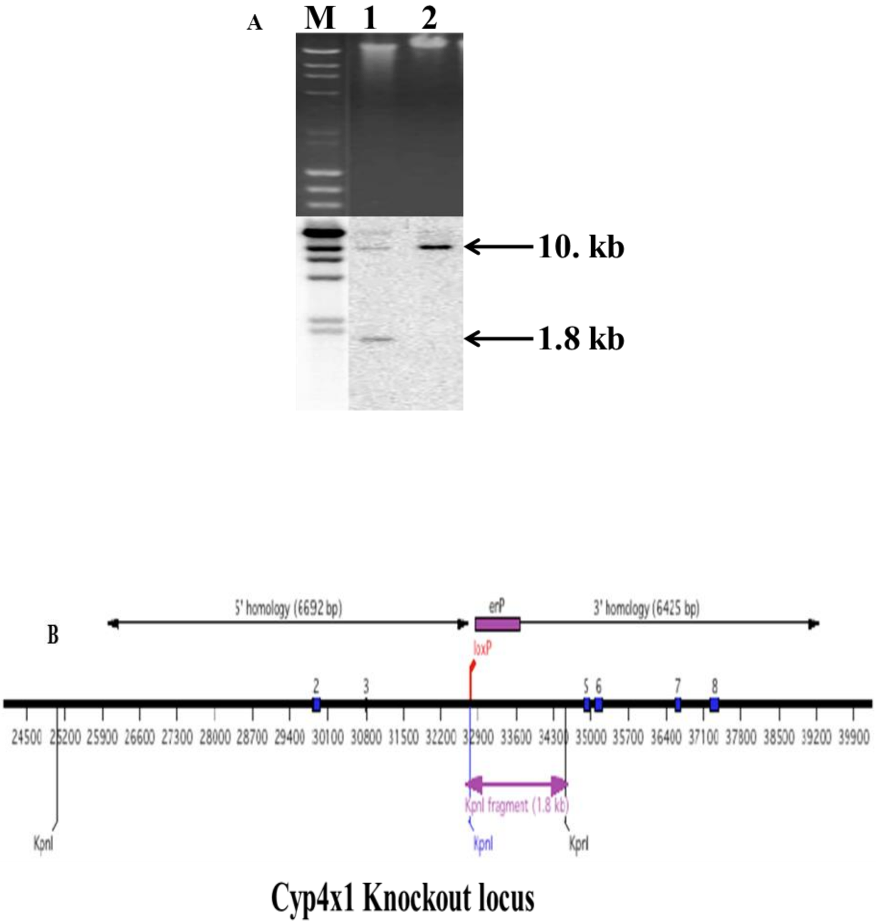

S4 Fig: Genotyping of of Cyp4x1$^{WT/KO/Cre}$ mice by Southern blotting. Genomic DNA from the tail was digested with KpnI and probed with the enP probe. (A) The wild-type allele produces a band of 10.0 kb, the Flox allele produces a 4.3 kb band and the Cre mediated deletion allele (a knockout allele) produces a band of 1.8Kb (figure A, lane 1), lane 2 represents a wild-type allele. This membrane was re-probed with probes for Cre and Neo in order to confirm the genotype (data not shown). Figure B represents the details of the *KpnI* restriction map and the enP probe in the knockout allele.
