# Supplementary material for: Generation and phenotypic characterisation of a cytochrome P450 4x1 knockout mouse
Source: PLoS One. 2017 Dec 11;12(12):e0187959. doi: 10.1371/journal.pone.0187959 (PMC5724839; doi:10.1371/journal.pone.0187959)
Supplement: S5 Fig — (PDF) [file pone.0187959.s006.pdf]

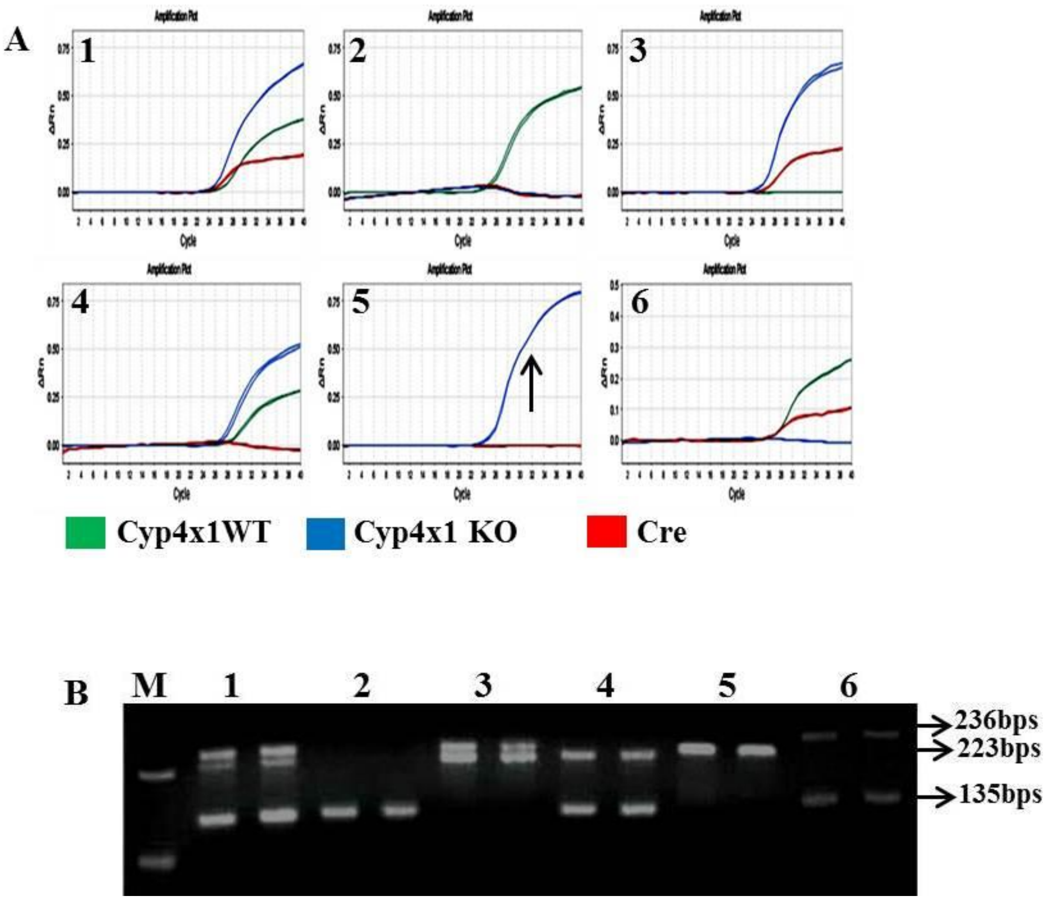

S5 Fig: Genotyping of Cyp4x1 KO mice.

A Three different probes were used in order to target the Cyp4x1 wild type allele (green), the Cyp4x KO allele (Blue) and the Cre allele (Red) in a multiplex qPCR reaction from genomic DNA. The sample in panel 4 represents the heterozygous knockout whilst panel 5 represents a homozygous Cyp4x1 knockout

B The genotypes were confirmed by agarose gel electrophoresis after running samples no 1-6 shown in fig 5A. Wild type primers produce a 135 bps amplicon (sample 1, 2, 4, 6) while the knockout primers produce a 223 bps amplicon (sample 1, 3, 4, 5). Cre primers produce a 236 bps amplicon (sample 1, 3 and 6). Lane 4 and 5 represent the heterozygous and homozygous KO mice.
